# Incidence, risk factors, and prognostic impact of regional lymph node metastasis in bone sarcoma: a population-based cohort study

**DOI:** 10.1093/jjco/hyaf096

**Published:** 2025-06-08

**Authors:** Hiroshi Kobayashi, Liuzhe Zhang, Koichi Okajima, Yusuke Tsuda, Toshihiko Ando, Toshihide Hirai, Akira Kawai, Sakae Tanaka

**Affiliations:** Department of Orthopaedic Surgery, The University of Tokyo, 7-3-1 Hongo, Bunkyo-ku, Tokyo 113-8655, Japan; Department of Orthopaedic Surgery, The University of Tokyo, 7-3-1 Hongo, Bunkyo-ku, Tokyo 113-8655, Japan; Department of Orthopaedic Surgery, The University of Tokyo, 7-3-1 Hongo, Bunkyo-ku, Tokyo 113-8655, Japan; Department of Orthopaedic Surgery, The University of Tokyo, 7-3-1 Hongo, Bunkyo-ku, Tokyo 113-8655, Japan; Department of Orthopaedic Surgery, The University of Tokyo, 7-3-1 Hongo, Bunkyo-ku, Tokyo 113-8655, Japan; Department of Musculoskeletal Oncology, Tokyo Metropolitan Cancer and Infectious Diseases Center, Komagome Hospital, 3-18-22 Honkomagome, Bunkyo-ku, Tokyo 113-8677, Japan; Department of Musculoskeletal Oncology and Rehabilitation Medicine, National Cancer Center Hospital, Rare Cancer Center, National Cancer Center Hospital, 5-1-1 Tsukiji, Chuo-ku, Tokyo 104-0045, Japan; Department of Orthopaedic Surgery, The University of Tokyo, 7-3-1 Hongo, Bunkyo-ku, Tokyo 113-8655, Japan

**Keywords:** bone sarcoma, lymphatic metastasis, prognosis, risk factors, survival analysis

## Abstract

**Background:**

This study aimed to investigate the risk factors and prognostic impact of regional lymph node metastasis (RLNM) in patients with bone sarcoma.

**Methods:**

This retrospective study analyzed data from a Japanese registry of patients with bone sarcoma (2006–19). Disease-specific overall survival was estimated using the Kaplan–Meier method. A Cox regression model was used to identify risk factors for RLNM and prognostic factors.

**Results:**

Among 5064 patients, 157 (3.1%) had RLNM. The incidence varied by histological subtype: 7.6% in Ewing sarcoma, 3.1% in osteosarcoma, 1.6% in chondrosarcoma, and 5.2% in undifferentiated pleomorphic sarcoma. Higher rates were observed in rare subtypes, including mesenchymal chondrosarcoma (12.9%) and dedifferentiated chondrosarcomas (10.3%). Risk factors for RLNM included older age, tumor size (>8 cm) (*P* = .02), distant metastasis at diagnosis (*P* < .0001), skip metastasis (*P* < .0001), and histological subtype (e.g. Ewing sarcoma and dedifferentiated chondrosarcoma). RLNM was associated with poor prognosis (HR 1.69, 95% CI: 1.35–2.1, *P* < .0001), with isolated RLNM conferring survival outcomes equivalent to those with distant metastasis. Among RLNM cases, skip metastasis was the only significant independent predictor of poor prognosis (HR 2.41, 95% CI: 1.35–4.30, *P* = .003).

**Conclusions:**

The incidence of RLNM in bone sarcomas varies by histological subtype. Risk factors include older age, tumor size, distant metastasis, skip metastasis, and histological subtype. Isolated RLNM has a prognosis comparable to that of distant metastases, and skip metastasis is a significant negative prognostic factor.

## Introduction

Bone sarcomas are rare mesenchymal malignancies with heterogeneous subtypes, among which osteosarcoma, chondrosarcoma, and Ewing sarcoma are the most common. The lungs are the primary site of metastasis via hematogenous spread [1,2], and numerous studies have investigated the risk factors for pulmonary metastasis in bone sarcomas [3–5]. In contrast, regional lymph node metastasis (RLNM) is rare, with only one large-scale study using the SEER database analyzing its incidence and risk factors [6]. However, the incidence of RLNM in ultra-rare bone sarcomas [7] remains unknown.

In soft tissue sarcomas (STS), RLNM is recognized as a poor prognostic factor [8–10]. Some reports suggest that patients with isolated RLNM (without distant metastasis), which is classified as N1M0 in TNM classification (TNM-UICC/AJCC 8th ed), have better survival than those with distant metastasis, although findings have been inconsistent [11,12]. However, the prognostic significance of isolated RLNM in bone sarcomas remains unclear.

Skip metastasis is defined by the American Joint Committee on Cancer as “two or more discontinuous lesions in the same bone” [13]. Patients with skip metastasis but no other metastases are classified as Stage III, whereas those with locally contained disease are classified as Stage II, as skip metastasis is considered an independent poor prognostic factor [13]. However, some studies have reported similar survival outcomes between patients with and without skip metastasis [14,15]. To date, no study has investigated the relationship between RLNM and skip metastases in bone sarcoma.

To address these gaps, this study aims to determine the incidence and risk factors for RLNM in bone sarcomas, evaluate its prognostic significance, and assess the relationship between RLNM and skip metastases. By analyzing a national database, we seek to clarify the clinical impact of RLNM on disease outcomes and inform future staging and management strategies.

## Materials and methods

### Data source

This study utilized data from the Japanese Nationwide Bone and Soft Tissue Tumor Registry, a database established by the Japanese Orthopedic Association (JOA) to systematically collect information on bone and soft tissue tumors from JOA-certified hospitals. The registry includes demographic data, clinical and pathological tumor characteristics, treatment details, and prognostic outcomes at the latest follow-up. The methodology and structure of this database have been described previously [16]. Approval for this study was obtained from the Musculoskeletal Tumor Committee of the JOA [17] and the Institutional Review Board (IRB) of the JOA and our institution (approval number: 2024173NI). Given that all data were deidentified, the requirement for informed consent was waived by the IRB.

**Table 1:** Clinical characteristics of patients with and without RLNM.

	RLNM (+)	%	RLNM (−)	%	*P*-value
**Age**					.03
0–40	56	35.6	2156	43.9	
41–65	43	27.4	1414	28.8	
66–	58	36.9	1337	27.3	
**Sex**					.34
Female	62	39.5	2127	43.3	
Male	95	60.5	2780	56.7	
**Size**					<.0001
<8	45	30.2	2316	48.6	
≧8	104	69.8	2446	51.4	
**Location**					.17
Head and neck	4	2.8	34	0.7	
Upper extremity	17	11.8	545	11.4	
Lower extremity	65	45.1	2305	48.2	
Trunk	58	40.3	1897	39.7	
**Grade**					<.0001
High grade	140	89.2	3350	68.3	
Low grade	12	7.6	1308	26.7	
Unknown	5	3.2	249	5.1	
**Distant metastasis**					<.0001
+	59	37.6	585	11.9	
−	97	61.8	4297	87.6	
Unknown	1	0.6	25	0.5	
**Skip metastasis**					<.0001
+	29	18.5	186	3.8	
−	121	77.1	4677	95.3	
Unknown	7	4.5	44	0.9	
**Surgery for primary tumor**					<.0001
+	87	55.4	3515	71.6	
−	70	44.6	1372	28.0	
Unknown	0	0.0	20	0.4	
**Radiotherapy**					<.0001
+	77	49.0	1057	21.5	
−	80	51.0	3830	78.1	
Unknown	0	0.0	20	0.4	
**Chemotherapy**					<.0001
+	103	65.6	2263	46.1	
−	54	34.4	2631	53.6	
Unknown	0	0.0	13	0.3	

Abbreviation: RLNM, regional lymph node metastasis.

**Table 2:** Rate of RLNM by histological subtype.

Histology	Total	Positive	%
Mesenchymal chondrosarcoma	31	4	12.9
Dedifferentiated chondrosarcoma	97	10	10.3
Fibrosarcoma	36	3	8.3
Ewing sarcoma	357	27	7.6
Undifferentiated pleomorphic sarcoma	307	16	5.2
Osteosarcoma	2095	65	3.1
Malignant giant cell tumor	36	1	2.8
Leiomyosarcoma	86	2	2.3
Others	207	4	2
Chondrosarcoma	1314	21	1.6
Hemangioendothelioma	64	1	1.6
Chordoma	388	3	0.8
Clear cell sarcoma	46	0	0

**Table 3:** Risk factors for RLNM in patients with bone sarcoma.

	Odds ratio	*P*-value
**Age**		
0–40	1	
41–65	2.1	.002
66–	2.92	<.0001
**Sex**		
Female		
Male		
**Size**		
<8	1	
≧8	1.54	.02
**Location**		
Head and neck		
Upper extremity		
Lower extremity		
Trunk		
**Grade**		
High grade	1.76	.12
Low grade	1	
**Distant metastasis**		
+	2.22	<.0001
−	1	
Unknown		
**Skip metastasis**		
+	3.35	<.0001
−	1	
**Histological subtype**		
Dedifferentiated chondrosarcoma	4.01	.002
Undifferentiated pleomorphic sarcoma	1.97	.07
Ewing sarcoma	4.94	<.0001
Mesenchymal chondrosarcoma	6.28	.01
Osteosarcoma	1.91	.04
Chordoma	0.5	.28
Fibrosarcoma	4	.05
Leiomyosarcoma	0.93	.92
Chondrosarcoma	1	

### Patient selection and data extraction

Patients diagnosed with bone sarcoma between 2004 and 2019 were identified in the registry. Eligible patients had histologically confirmed bone sarcoma with sufficient prognostic data for analysis. Patients diagnosed with intermediate or benign tumors, including giant cell tumors of the bone and desmoplastic fibromas, were excluded. Cases with missing information on regional lymph node metastases were also excluded. A total of 5064 patients met the eligibility criteria. For each patient, data were extracted on demographic characteristics (age and sex), tumor characteristics (tumor size, primary location, histological subtype, and tumor grade), clinical status (presence of lymph node metastasis at initial diagnosis [synchronous] or after surgery [metachronous]), and therapeutic interventions (surgical and non-surgical treatments). Age was categorized into three groups: <40 years, 40–64 years, and ≥65 years. Tumor location was classified into four anatomical regions: upper extremity, lower extremity, head and neck, and trunk.

Disease-specific overall survival (DOS) was defined as the time from diagnosis to disease-related death. Patients who remained alive at their last follow-up or were lost to follow-up were censored at their last recorded visit. To ensure adequate follow-up, patients with <6 months of follow-up were excluded.

### Statistical analysis

Comparisons between categorical and continuous variables were conducted using Fisher’s exact test and the Wilcoxon test, respectively. Survival analysis was performed using the Kaplan–Meier method, and cumulative survival rates were compared using the log-rank test. A *P* < .05 was considered statistically significant. To assess potential prognostic factors, univariate and multivariate analyses were conducted using the Cox proportional hazards model. Results are reported as adjusted hazard ratios (HRs) with 95% confidence intervals (CI) and corresponding *P*-values (two-sided alpha = 0.05). All statistical analyses were performed using JMP Pro 18 software (SAS Institute Inc., Cary, NC, USA).

**Figure 1:**
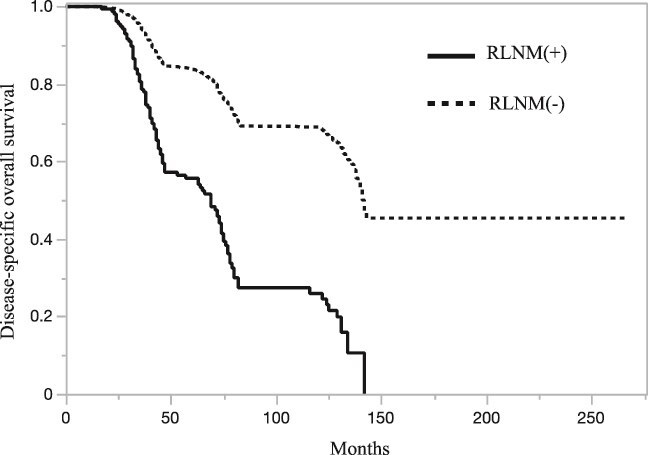
DOS in patients with RLNM.

**Table 4:** Multivariate analysis of disease-specific overall survival in patients with bone sarcoma.

	Univariate analysis			Multivariate analysis		
	Hazard ratio	95% CI	*P*-value	Hazard ratio	95% CI	*P*-value
**Age**						
0–40	1			1		
41–65	1.12	0.98–1.29	.1	1.79	1.53–2.09	<.0001
66–	1.6	1.4–1.82	<.0001	2.53	2.14–2.97	<.0001
**Sex**						
Female	1					
Male	1.11	0.99–1.24	.07			
**Size**						
<8	1			1		
≧8	1.89	1.67–2.13	<.0001	1.36	1.2–1.54	<.0001
**Location**						
Head and neck	1					
Upper extremity	0.61	0.29–1.25	.18			
Lower extremity	0.83	0.41–1.67	.6			
Trunk	1.1	0.55–2.22	.78			
**Grade**						
High grade	5.7	4.56–7.12	<.0001	2.73	2.11–3.53	<.0001
Low grade	1			1		
**Distant metastasis**						
+	4.3	3.82–4.48	<.0001	2.46	2.15–2.82	<.0001
−	1			1		
**Skip metastasis**						
+	2.87	2.38–3.45	<.0001	1.5	1.22–1.84	<.0001
−	1			1		
**RLNM**						
+	3.51	0.23–0.35	<.0001	1.69	1.35–2.1	<.0001
−	1			1		
**Histological subtype**						
Dedifferentiated chondrosarcoma	6.48	4.8–8.74	<.0001	2.33	0.68–3.24	<.0001
Undifferentiated pleomorphic sarcoma	3.86	3.05–4.9	<.0001	1.59	1.22–2.06	.0005
Ewing sarcoma	3.27	2.58–4.13	<.0001	2.35	1.77–3.11	<.0001
Mesenchymal chondrosarcoma	3.42	1.9–6.16	<.0001	2.58	1.38–4.82	.003
Osteosarcoma	2.6	2.18–3.11	<.0001	1.96	1.59–2.41	<.0001
Chordoma	0.72	0.5–4.66	.08	0.67	0.46–0.97	.03
Fibrosarcoma	2.38	1.22–4.66	.01	1.19	0.6–2.34	.62
Leiomyosarcoma	3.18	2.06–4.88	<.0001	1.87	1.2–2.9	.006
Chondrosarcoma	1					

Abbreviation: RLNM, regional lymph node metastasis.

**Table 5:** Prognostic impact of isolated RLNM.

Histological subtype	N0M0				N1M0		N0M1				N1M1			
	Number	Hazard ratio	95% CI	*P*-value	Number	Hazard ratio	Number	Hazard ratio	95% CI	*P*-value	Number	Hazard ratio	95% CI	*P*-value
Ewing sarcoma	262	0.54	0.17–1.72	.3	6	1	76	1.24	0.38–3.97	.72	12	1.36	0.36–5.13	.65
Osteosarcoma	1730	0.64	0.24–1.71	.37	9	1	320	2.44	0.91–6.56	.08	33	4.63	1.57–13.72	.006
Chondrosarcoma	1258	0.05	0.01–0.19	<.0001	4	1	42	0.42	0.1–1.79	.24	3	1.69	0.28–10.1	.57
ALL	4371	0.33	0.19–0.58	.0001	23	1	594	1.39	0.78–2.47	.26	50	1.92	1.01–3.68	.047

**Figure 2:**
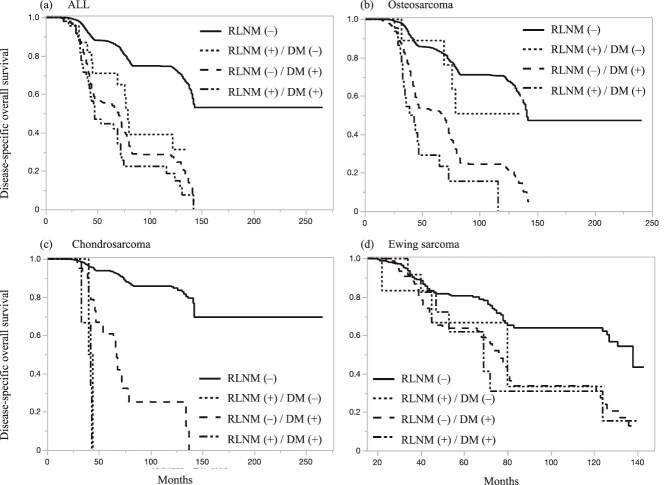
Impact of regional lymph node metastasis on disease-specific overall survival by histological subtype. (a) All cases, (b) osteosarcoma, (c) chondrosarcoma, and (D) Ewing sarcoma.

## Results

### Patient characteristics and histological subtypes

Among 5064 patients with bone sarcoma, 157 (3.1%) had RLNM, including 74 with synchronous RLNM and 83 with metachronous RLNM. Table 1 summarizes the clinical characteristics of patients with and without RLNM. The mean age of patients with RLNM was 54 years (range: 4–90 years), which was significantly higher than that of patients without RLNM (mean: 45 years, range: 0–95 years, *P* = .01). Patients with RLNM had significantly larger, high-grade tumors. Distant metastasis at the time of diagnosis was more frequently observed in patients with RLNM than in those without RLNM (37.6% vs. 11.9%; *P* < .0001). Similarly, skip metastases were significantly more common in patients with RLNM than in those without RLNM (18.5% vs. 3.8%; *P* < .0001). Compared with patients without RLNM, those with RLNM were less likely to undergo surgery for the primary tumor (55.4% vs. 71.6%, *P* < .0001) and more likely to receive radiotherapy (49.0% vs. 21.5%; *P* < .0001) and chemotherapy (65.6% vs. 46.1%; *P* < .0001).

Table 2 presents the RLNM positive rates by histological subtype. Among bone sarcomas with over 100 cases, Ewing sarcoma (7.6%) and undifferentiated pleomorphic sarcoma (5.2%) had the highest RLNM rates, whereas chondrosarcoma (1.6%) and chordoma (0.8%) had the lowest rates. Among ultra-rare subtypes, mesenchymal chondrosarcoma (12.9%, 4 of 31) and dedifferentiated chondrosarcoma (10.3%, 10 of 97 cases) exhibited relatively high RLNM rates.

Table 3 presents the results of multivariate analysis for RLNM risk factors. Older age (*P* < .0001), tumor size >8 cm (*P* = .02), distant metastasis at diagnosis (*P* < .0001), and skip metastasis (*P* < .0001) were all independently associated with RLNM. Regarding histological subtypes, dedifferentiated chondrosarcoma (*P* = .002), Ewing sarcoma (*P* < .0001), mesenchymal chondrosarcoma (*P* = .01), and osteosarcoma (*P* = .04) were identified as significant risk factors for RNLM.

### Prognostic impact of RLNM

The median follow-up period was 66 months (range: 13–266 months). The 3-year and 5-year DOS rates for patients with RLNM were 78.6% and 55.7%, respectively. Patients with RLNM had significantly poorer survival than those without RLNM (median DOS: 69 months [95% CI: 47–74] vs. 142 months [95% CI: 140–censored], *P* < .0001; Fig. 1). Multivariate analysis confirmed RLNM as an independent predictor of poor prognosis (HR 1.69, 95% CI: 1.35–2.1, *P* < .0001; Table 4). To further evaluate the prognostic impact of RLNM and distant metastasis, patients were divided into four groups based on the presence or absence of RLNM and distant metastasis. Table 5 and Fig. 2 illustrate these prognostic comparisons. Among all patients with bone sarcoma, isolated RLNM had a prognosis similar to that of distant metastasis. However, the prognostic impact of isolated RLNM varied by histological subtype, though the number of cases was limited. In osteosarcoma, patients with isolated RLNM had a prognosis comparable to that of patients without RLNM or distant metastasis (HR 0.64, *P* = .37), but their survival was significantly better than that of patients with distant metastasis (HR 2.44, *P* = .08) or those with both RLNM and distant metastasis (HR4.63, *P* = .0006). In Ewing sarcoma, the prognostic impact of isolated RLNM was equivalent to that of distant metastasis, whereas in chondrosarcoma, RLNM was associated with particularly poor survival outcomes compared to patients without RLNM or distant metastasis.

### Prognostic factors in patients with RLNM

Table 6 presents the prognostic factors for patients with RLNM. In univariate analysis, distant metastasis, skip metastasis, and mesenchymal chondrosarcoma were associated with poor prognosis. Multivariate analysis identified skip metastasis as the only independent predictor of poor prognosis (HR 2.41, 95% CI: 1.35–4.30, *P* = 0.003).

**Table 6:** Prognostic factor in patients with RLNM.

	Uni-variate analysis			Multi-variate analysis		
	Hazard ratio	95% CI	P-value	Hazard ratio	95% CI	P-value
Age						
0-40	1					
41–65	1.61	0.99–2.62	0.05			
66–	1.48	0.92–2.37	0.1			
Sex						
Female	1					
Male	1.04	0.7–1.55	0.86			
Size						
<8	1					
≧8	1.41	0.9–2.21	0.14			
Location						
Head and neck	1					
Upper extremity	0.43	0.09–1.98	0.28			
Lower extremity	0.56	0.13–2.34	0.43			
Trunk	0.59	0.14–2.47	0.47			
Grade						
High grade	2.09	0.85–5.14	0.11			
Low grade	1					
Distant metastasis						
+	1.71	1.16–2.53	0.007	1.26	0.77–2.02	0.37
−	1			1		
Skip metastasis						
+	2.87	1.81–4.57	<0.0001	2.41	1.35–4.30	0.003
−	1			1		
Timing of RLNM						
synchronous	1					
metachronous	0.89	0.61–1.32	0.57			
Histological subtype						
Dedifferentiated chondrosarcoma	1.67	0.64–4.36	0.29	1.59	0.58–4.36	0.37
Undifferentiated pleomorphic sarcoma	1.3	0.55–3.1	0.55	1.02	0.42–2.46	0.97
Ewing sarcoma	1.22	0.54–2.77	0.63	1.14	0.49–2.66	0.75
Mesenchymal chondrosarcoma	6.14	1.62–23.2	0.008	2.65	0.67–10.56	0.17
Osteosarcoma	1.84	0.9–3.75	0.09	1.58	0.76–3.27	0.22
Chordoma	0.97	0.21–4.51	0.97	1.03	0.22–4.83	0.97
Fibrosarcoma	1.48	0.32–6.9	0.61	1.68	0.36–7.83	0.51
Leiomyosarcoma	3.39	0.43–27.01	0.25	4.03	0.5–32.18	0.19
Chondrosarcoma	1			1		

## Discussion

RLNM is rare in bone sarcomas, occurring in 3.1% of cases in this study, consistent with previous reports (2.9%) [6]. Among common bone sarcomas, Ewing sarcoma exhibited the highest RLNM rate, aligning with prior findings [6,18–20]. Additionally, our study is the first to report high RLNM rates in ultra-rare sarcomas, specifically dedifferentiated chondrosarcoma (10.3%) and mesenchymal chondrosarcoma (12.9%). By analyzing a large dataset, we identified several risk factors for RLNM, including older age, larger tumor size, distant metastasis at diagnosis, and histological subtype. Notably, RLNM was associated with significantly worse prognosis, with isolated RLNM conferring a survival outcome similar to that of distant metastasis. Furthermore, skip metastasis emerged as a risk factor for RLNM and an independent prognostic indicator of poor survival in patients with RLNM.

The RLNM rate was similar to that reported in prior large-scale analyses, further supporting the histology-dependent variability in RLNM incidence. Specifically, the rates of RLNM in this study versus previous reports were 7.6% vs. 6.0% in Ewing sarcoma, 3.1% vs. 2.5% in osteosarcoma, and 1.6% vs. 1.1% in chondrosarcoma [6]. These findings are consistent with those of previous reports that analyzed RLNM incidence by histological subtype [18–20]. Few studies have assessed risk factors for RLNM in bone sarcomas. Dong et al. analyzed 311 of 10 641 patients with RLNM and identified male sex, primary tumor location (skull, face, and mandible), tumor type (Ewing sarcoma vs. osteosarcoma), distant metastasis, and tumor size (>12 cm vs. <6 cm) as risk factors [6]. In contrast, our study did not find tumor location or sex to be associated with RLNM. However, we confirmed that tumor size, distant metastasis, and histological subtype were significant risk factors, which is consistent with a previous report [6]. Notably, our study identified skip metastasis as the most significant risk factor for RLNM, a relationship that has not been previously reported. This novel finding suggests that skip metastasis may serve as an early indicator of lymphatic spread, underscoring the need for further investigation. These results could aid physicians in detecting RLNM, which remains rare in bone sarcomas.

Our findings also confirm that RLNM is an independent predictor of poor prognosis. Among all patients with RLNM, 60% had isolated RLNM, and their survival outcomes were comparable to those of patients with distant metastasis. In STS, several reports have suggested that patients with isolated RLNM have better survival than those with distant metastases, although findings remain inconclusive [8–12]. To our knowledge, this is the first study to analyze the prognostic impact of isolated RLNM in bone sarcoma. Given the rarity of RLNM in these tumors, our findings suggest that its presence indicates aggressive disease behavior similar to distant metastasis. However, due to the small number of cases with isolated RLNM, further studies with larger cohorts are warranted to validate these findings.

Additionally, our study highlights skip metastasis as a significant risk factor for RLNM and a poor prognostic factor for patients with RLNM. In our cohort, skip metastases were observed in 4.3% of all patients and in 18.5% of patients with RLNM. The reported incidence of skip metastasis in osteosarcoma ranges from 1.4% to 25% [14,15,21,22], with variations likely attributed to differences in imaging modalities and detection methods. However, no prior studies have identified skip metastasis as a risk factor for RLNM. Our findings suggest that skip metastasis may serve as a marker of aggressive tumor behavior. The prognostic impact of skip metastases in osteosarcoma remains debated [6,14,15,22], with conflicting reports on its effect on survival. Mechanistically, skip metastasis is thought to result from hematogenous tumor spread. In papillary thyroid cancer, multifocal primary lesion is reported to be risk factor of lymph node metastasis [23,24]. However, in early-stage gastric cancer, conflicting evidence that synchronous multiple primary lesions are associated with lymph node metastasis [25,26]. Whether multiple primary lesions reflect aggressive nature of tumor depends on the cancer type. Our observation that skip metastasis is associated with an increased likelihood of RLNM in bone sarcoma suggests that tumors exhibiting skip metastases may have a higher metastatic potential, warranting further investigation into the biological mechanisms linking these two metastasis patterns.

This study has several limitations that should be considered when interpreting the results. First, the sample size was relatively small, particularly for patients with isolated RLNM, which may have limited the statistical power of subgroup analyses. Second, the screening and diagnosis of RLNM and skip metastasis varied between institutions, potentially introducing differences in detection rates. Third, histological diagnoses were institution-dependent, although all participating centers specialize in bone and soft tissue tumors, which likely mitigates classification inconsistencies. Despite these limitations, this study represents one of the largest analyses of RLNM in bone sarcomas and is the first to assess the incidence of RLNM in ultra-rare sarcomas, evaluate the prognostic impact of isolated RLNM, and investigate the relationship between RLNM and skip metastasis.

In conclusion, we determined the incidence of RLNM in bone sarcomas, identified associated risk factors, and evaluated its prognostic impact. RLNM rates varied by histological subtype, with Ewing sarcoma exhibiting a relatively high incidence among common bone sarcomas, and mesenchymal chondrosarcoma and dedifferentiated chondrosarcoma showing high RLNM rates among ultra-rare sarcomas. Older age, large tumor size, distant metastasis, skip metastasis, and histological subtype were significant risk factors for RLNM. The prognostic impact of RLNM was similar to that of distant metastasis. Furthermore, skip metastasis was not only a risk factor for RLNM but also an independent predictor of poor prognosis in patients with RLNM. These findings provide clinically relevant insights for identifying high-risk patients and tailoring management strategies. Future prospective studies should explore the underlying mechanisms linking skip metastasis to RLNM and assess whether enhanced imaging techniques could improve RLNM detection and prognosis. In addition, optimal treatment for RLNM, whether negative margin resection or adjuvant radiotherapy is needed or not, should be explored.

## Data Availability

The data that support the findings of this study are available from the corresponding author, [HK], upon reasonable request.
